# Actinomycetoma distal radius mimic osteosarcoma: A case report of a rare presentation

**DOI:** 10.1002/ccr3.7618

**Published:** 2023-06-26

**Authors:** Hassan Elbahri, Adnan Ayman Mohammed Adnan Alnaser, Sawsan A. M. Babiker, Alaa Hatim Ameer Mohamed

**Affiliations:** ^1^ Department of orthopedic, Faculty of Medicine International University of Africa Khartoum Sudan; ^2^ Orthopedic specialist, Ibrahim Malik Teaching Hospital Khartoum Sudan; ^3^ Department of Pathology, Faculty of Medicine University of Khartoum Khartoum Sudan

**Keywords:** actinomycetoma, osteomyelitis, osteosarcoma, wide local excision

## Abstract

**Key Clinical Message:**

Actinmomycetma is a granulomatous infection with a presentation was very similar to osteosarccoma. Multidisciplinary team and triple assessments are extremely important to prevent misdiagnosis, surgical treatment in combination with medical treatment followed by regular clinical and radiological follow‐up can be a limb‐saving procedure in such cases.

**Abstract:**

Various conditions may mimic osteosarcoma. The differential diagnosis of osteosarcoma is broad; Includes tumors, infection, trauma, and inflammatory processes arising from the musculoskeletal system. A proper history, examination, diagnostic imaging studies, and pathological analysis are essential to establish a precise diagnosis. This case report serves to illustrate the significance of recognizing the similarities between these two lesions and other rare features that will help to differentiate between actinomycetoma and osteosarcoma, to prevent late or misdiagnosis.

## 1 INTRODUCTION

Osteosarcoma is the commonest primary malignant bone tumor of unintelligible origin, with a peak age of 10–19 years old.^1^ The distal radius is a rare site for osteosarcoma; it was reported less than <1%.^2^ Among all the bone tumors, osteosarcoma has the widest variety in clinical presentation, imaging findings, morphological, and histological features. The mainstream treatment is a combination of chemotherapy and surgical treatment which improved the overall prognosis in the last two decades.^3^, ^4^ However, mycetoma is a chronic cutaneous and subcutaneous painless swelling caused by two major types of pathogens, either bacterial type (actinomycotic) or fungus type (eumycotic).^5^ It is the most commonly neglected disease of humans in tropical and subtropical areas among low socioeconomic individuals.^5^, ^6^ It involves the skin and connective tissue. Painless destructive nature, late presentation, poor response to treatment, and high recurrence rate are characteristic features of this disease. Among all the endemic countries, Sudan has the highest incidence.^6^, ^7^, ^8^ Chronic inflammatory granuloma, various deformities, disabilities, and high morbidity rate are the commonest known complications of mycetoma, the disease can be potentially limb‐threatening in its late stage.^9^, ^10^ Clinically, mycetoma starts as a small painless lump that increases in size gradually, forming multiple sinuses with seropurulent discharge, and eventually multicolored grains appear.^11^ Meticulous clinical assessment and radiological and histopathological analysis of such lesions are important for a precise diagnosis and management, especially when dealing with unclear osteoarticular lesions. Management of mycetoma predominantly depends on the etiological agent, site, and extent of the disease.^12^ In our case, we report a diagnostic approach and management plan after years of local experience to improve the outcome and prevent complications and their sequelae on the functional status.

## 2 CASE PRESENTATION

**FIGURE 1:**
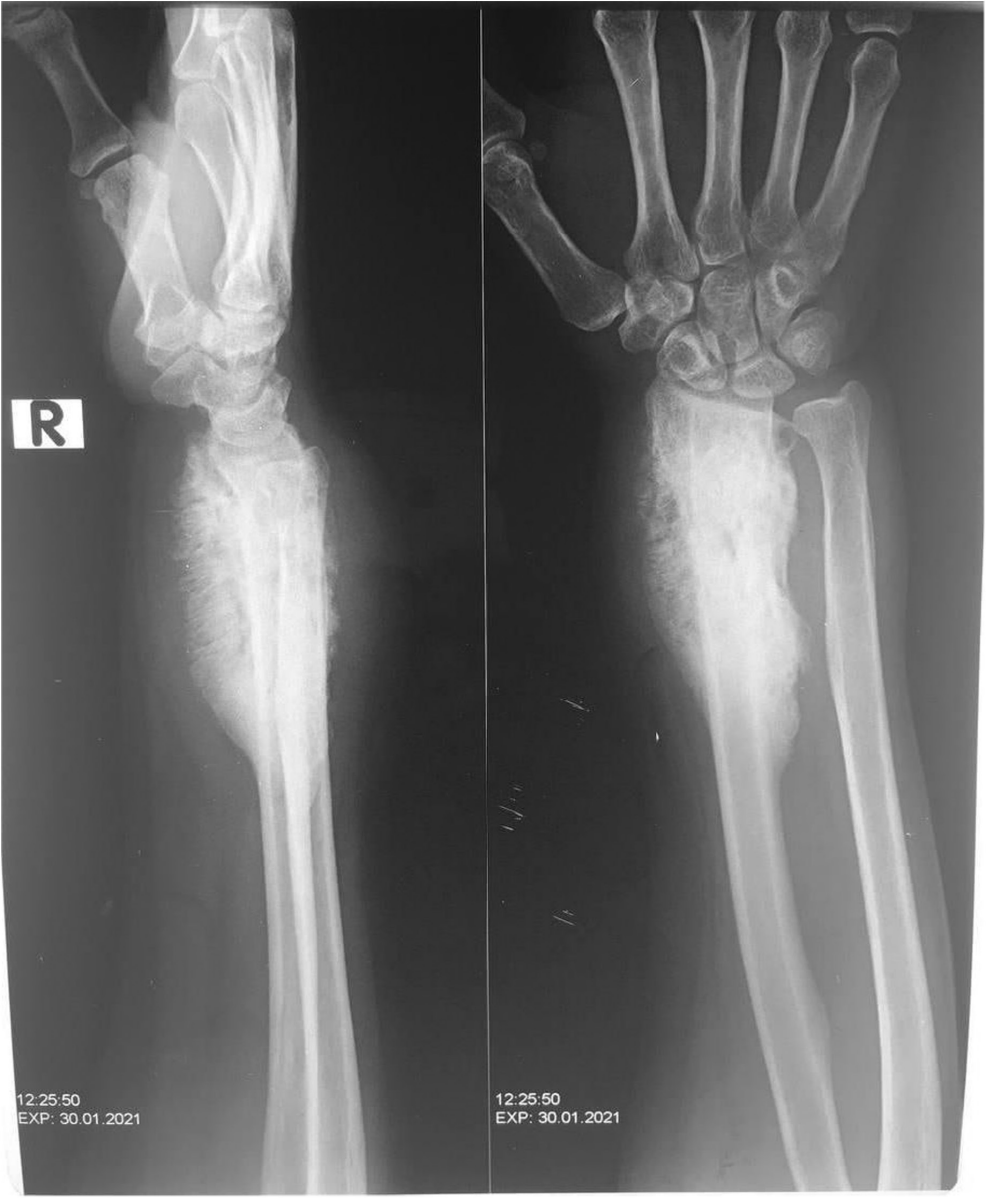
Preoperative X‐ray AP and lateral view of Rt forearm demonstrate mixed lytic and sclerotic bone lesion with sunburst periosteal reaction and soft tissue swelling.

**FIGURE 2:**
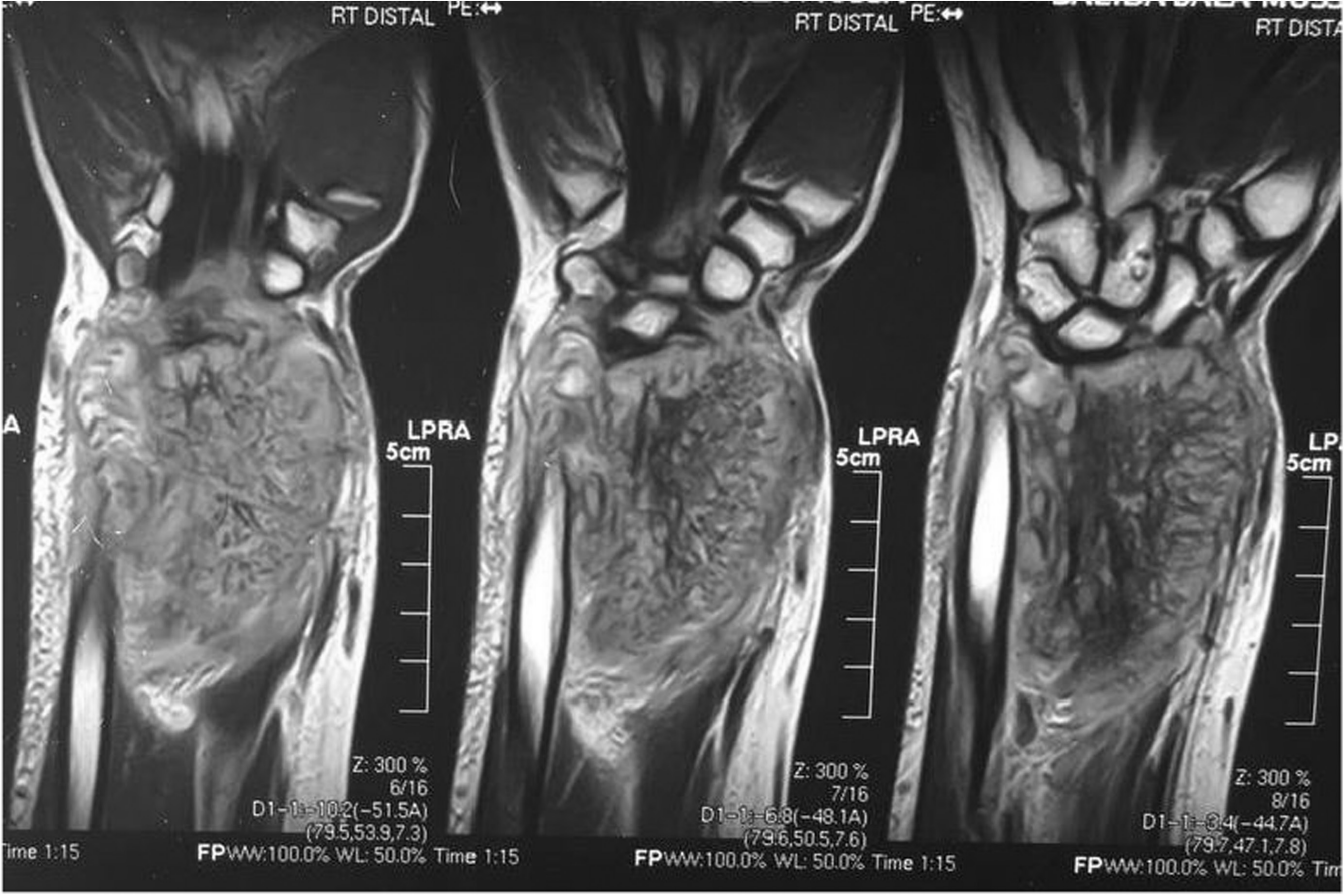
Preoperative MRI coronal view of Rt forearm shows right distal forearm swelling (enhancement) incasing distal neurovascular bundle, pretumor edema, and no skip lesions.

**FIGURE 3:**
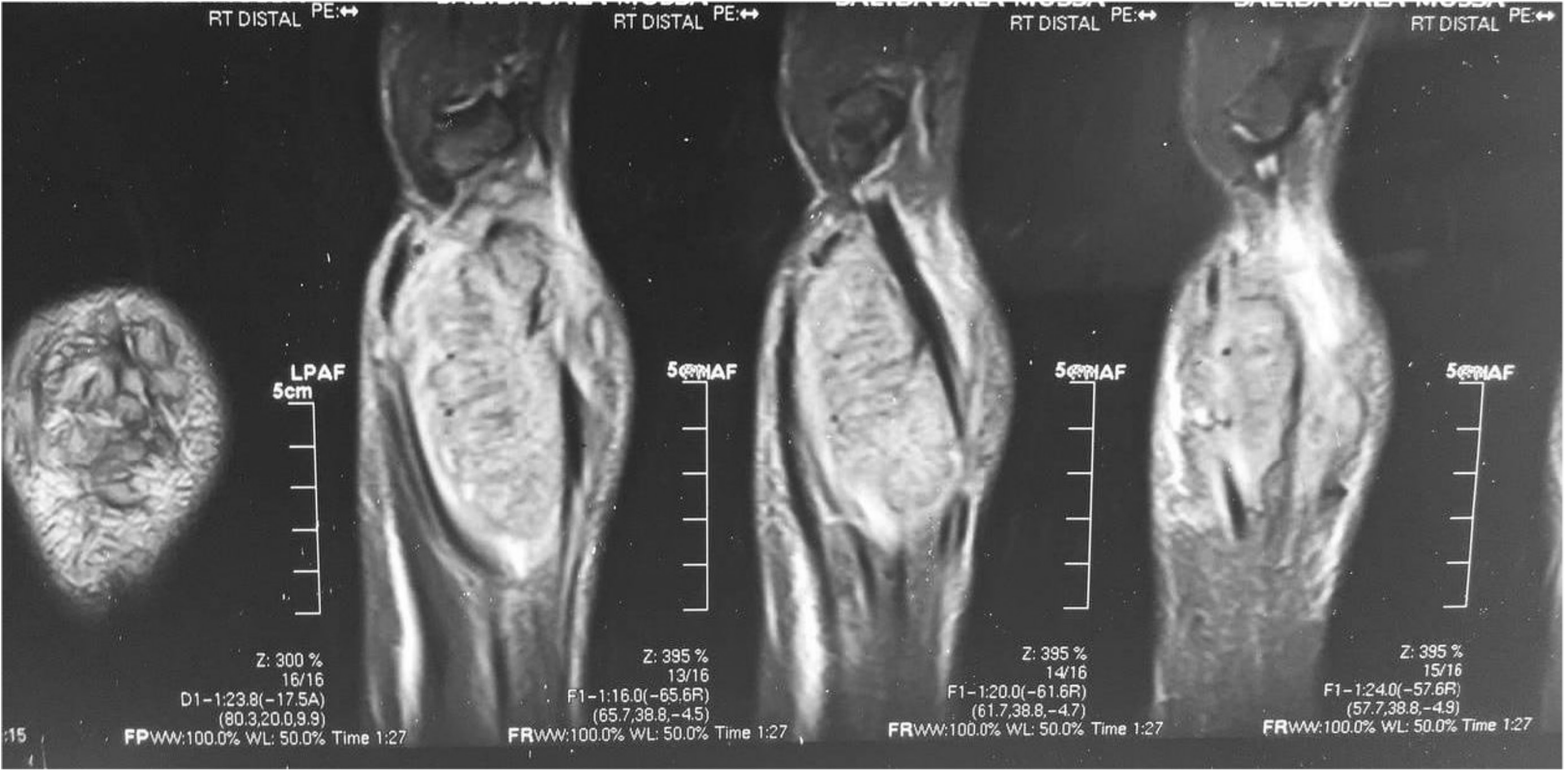
Preoperative MRI sagittal view of Rt forearm shows right distal forearm swelling (enhancement) incasing distal neurovascular bundle, pretumor edema, and no skip lesions.

**FIGURE 4:**
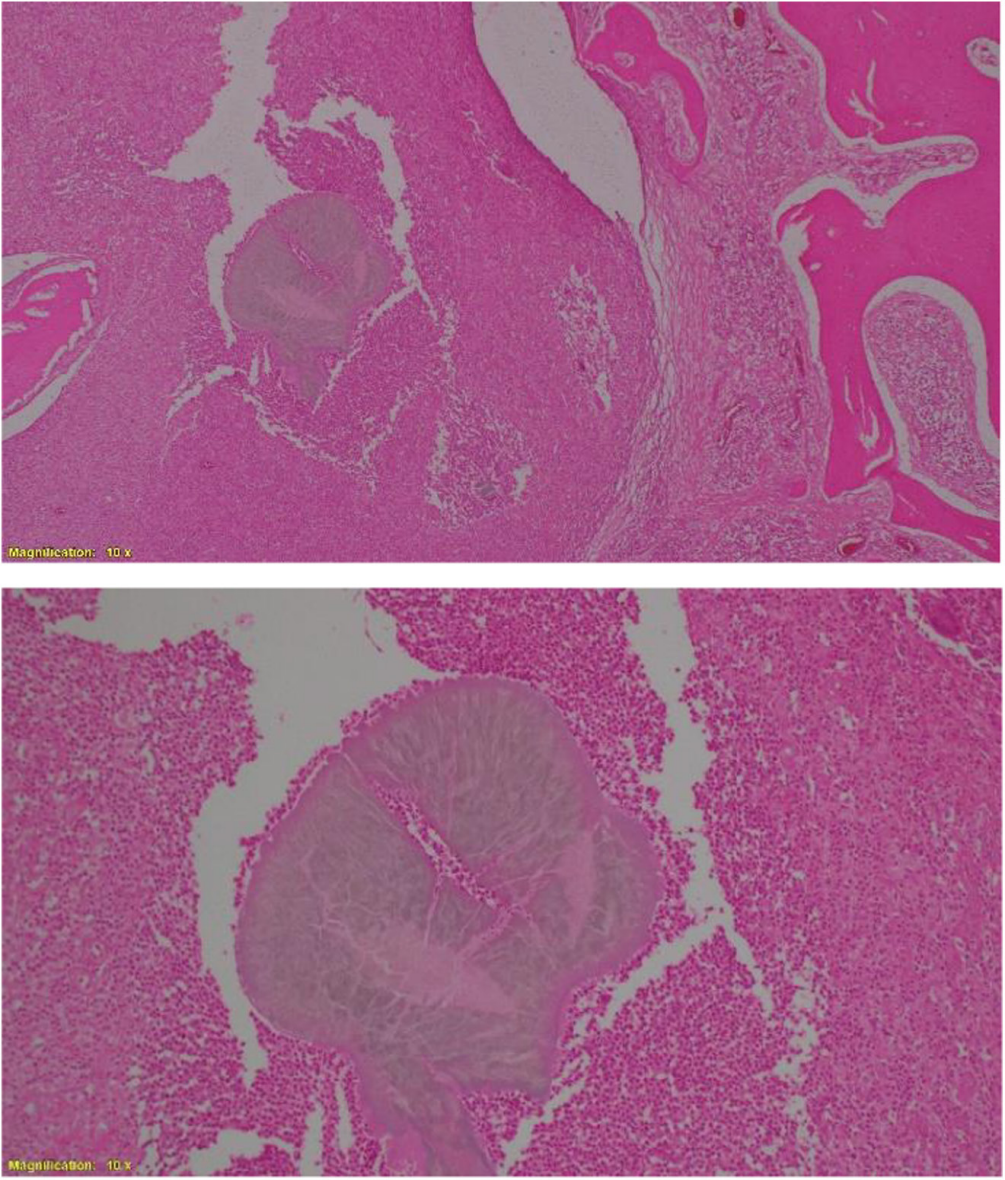
Shows bone trabeculae surrounding a grain of streptomyces somaliensis (above) x10HPF. The grain is surrounded by neutrophil microabscesses. There is minimal granulomatous inflammation (below) X20HPF.

A 40‐year‐old female farmer from Sudan was referred to our orthopedic oncology clinic at Ibrahim Malik Teaching Hospital—Sudan, complaining of chronic right distal forearm pain, swelling, and numbness for 2 years, it started with a small painless lump that over some time increased in size gradually, recently her quality of life and functional status had been massively affected due to increasing pain intensity, increase symptoms anguish and constant decrease in range of motion at the wrist joint. The patient was ill but not pale, with no loss of weight neither constitutional symptoms were noted, and vital signs were within normal range. No Sinuses were noted throughout the presentation. Conventional X‐ray shows a right distal radius mixed lytic and sclerotic bone lesion with sunburst periosteal reaction and soft tissue swelling (Figure 1). Initially, she was suspected of osteosarcoma distal radius, thus she was referred. No advanced imaging was requested, or biopsies were taken. Since then the deterioration in her general condition had been growing steadily. Painful activities of daily living agonized the patient. Swelling and limitation of movement on the affected side had been worsening dramatically. Furthermore, she ended up using daily painkillers for a long time. On clinical examination, there was obvious right distal forearm swelling and tenderness mainly at the radial and dorsal side of the forearm, the mass was irregular in shape, hard in consistency measured about 7*3 cm, with mild temperatures asymmetry compared with adjacent skin, no sinuses were identified lymphatic streaks. Hematological investigations were unremarkable, an X‐ray revealed the same findings, and an MRI of the right wrist showed right distal forearm swelling (enhancement), encasing distal neurovascular bundle, pretumor edema, and no skip lesions. (Figures 2, 3). CT chest and chest X‐ray were normal, with no evidence of metastases. The decision was to take a core biopsy under general anesthesia. The histopathological analysis reported multiple grains of streptomyces somaliensis, a causative agent of actinomycetoma (Figure 4). Secondly, a wide local excision and fixation were done and a second biopsy was taken, confirming the diagnosis.

**FIGURE 5:**
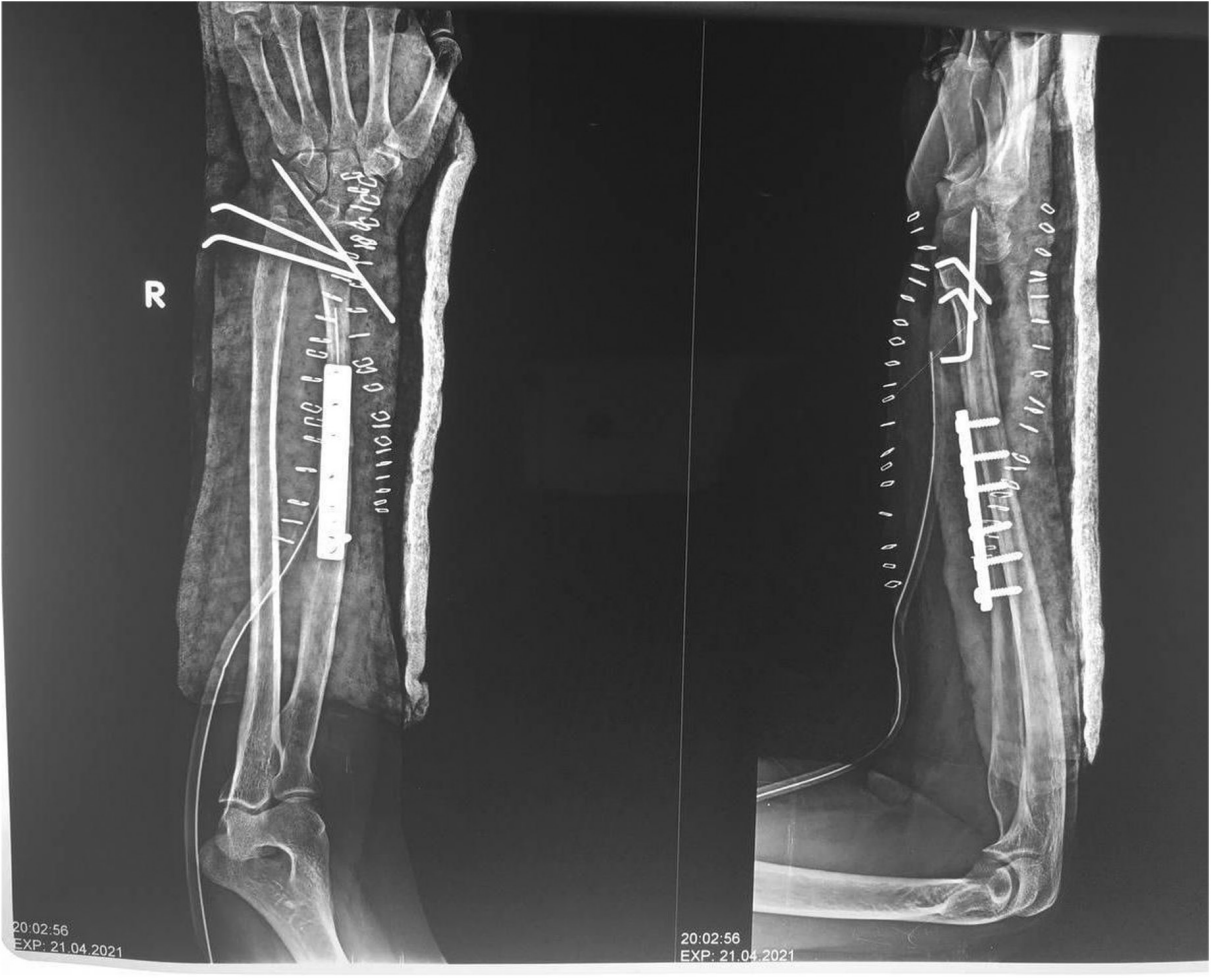
Postoperative X‐ray with anteroposterior and lateral views of the right forearm after the operation, showing a wide local excision and fixation with plate and K wires.

After 2 months of operation, the patient's condition improved as shown in the X‐ray (Figure 5).

## 3 DISCUSSION AND CONCLUSION

Actinomycetoma osteomyelitis radius is a very rare condition to be reported. Destructive bone patterns by mycetoma species, in situ grains formation, and the unequivocal distinction between actinomycetoma, pain, or numbness at late stages are extremely rare findings to be seen. The unique sunburst ray appearance can also be the source of significant diagnostic challenges. It is associated with a high risk of amputation. The initial painless nature of the lesion, low socioeconomic status, and low level of education are the most common reasons for late presentation among patients in Sudan.

The treatment of mycetoma predominantly depends on the type of infective agent, site, and extent of infection.^12^ In Sudan, amputation has just recently been a viable option for mycetoma treatment due to the disease's extraordinarily high recurrence rate, which makes other treatments ineffective.

Actinomycetoma (bacterial type) is usually treated with medications only as it shows a relative response to medical treatment in the early stages. A combination of aggressive surgical and medical (antifungal medicines) treatment for fungal type (eumycetoma) is the gold standard since medication resistance prevents exclusive medical treatment.^13^

Actinomycetoma infective agents are difficult to confirm. Therefore, accurate assessment should include proper clinical history and examination, radiological evaluation by an expert radiologist and orthopedic expert, pathological analysis of the affected area with a core needle biopsy, and immunohistochemistry to avoid problems of inadequate specimens commonly associated with incisional biopsy.^14^ The overall outcome can be optimized after precise identification of the causative organism and the extended post‐treatment follow‐up.

Unfortunately, surgical techniques were chosen in conjunction with medicinal treatment because of the patient's late presentation, aggressive invasion, and concern over a high recurrence rate. The surgical options for mycetoma treatment in Sudan include comprehensive local excision and amputation of the affected limb. For the best possible results during surgery, excision, and a bloodless field are crucial. To ensure a sufficient response, many Sudanese patients undergo numerous operations and lengthy medical regimens.^15^

The post‐treatment recurrence rate is high, ranging between 25% and 50%. Age, duration, site, extent of Involvement, and previous history of mycetoma recurrence are predictors of the overall outcome. Therefore, Surgical operation considers the best treatment option to minimize the risk of recurrence especially if done properly as mentioned above.^16^

Unfortunately, surgical intervention is associated with a high rate of morbidity and disability among mycetoma patients in Sudan. Postoperative follow‐up, physiotherapy of the nearby joints, and adherence to antibiotics regimen according to the protocol, are essential for better clinical and functional outcomes and to avoid permanent disabilities.^17^

In conclusion, actinomycetoma osteomyelitis radius is a rare condition. Clinicians in endemic areas must consider mycetoma osteomyelitis as a differential diagnosis when they are dealing with vague atypical musculoskeletal destructive lesions. Multidisciplinary team and triple assessments including clinical, radiological, and histopathological correlation are extremely important to prevent misdiagnosis, surgical treatment in combination with medical treatment followed by regular clinical and radiological follow‐up can be a limb‐saving procedure in such cases. Finally, treatment of mycetoma osteomyelitis cases should be prioritized according to the predictors of postoperative recurrence.^17^

## AUTHOR CONTRIBUTIONS

**Hassan Elbahri:** Conceptualization; data curation; formal analysis; writing – original draft. **Adnan Ayman Mohammed Adnan Alnaser:** Data curation; formal analysis; resources; software; visualization; writing – original draft; writing – review and editing. **Sawsan A. M. Babiker:** Data curation; writing – original draft. **Alaa Hatim ameer Mohamed:** Data curation; writing – original draft.

## FUNDING INFORMATION

This research received no specific grant from any funding agency in the public, commercial, or not‐for‐profit sectors.

## CONFLICT OF INTEREST STATEMENT

The authors declare that there are no conflicts of interest.

## ETHICS STATEMENT

Ethical approval was obtained from Sudan Medical Specialization Board. Both privacy and protection of the participant's files and information were of the highest priority. Written and verbal consents were taken from the participants.

## CONSENTS

Both written and verbal consents were taken from each patient.Written informed consent was obtained from the patient to publish this report in accordance with the journal's patient consent policy.

## Data Availability

The data that support the findings of this study are available from the corresponding author upon reasonable request.
